# Confirmation of independent introductions of an exotic plant pathogen of *Cornus* species, *Discula destructiva*, on the east and west coasts of North America

**DOI:** 10.1371/journal.pone.0180345

**Published:** 2017-07-26

**Authors:** Kristie Mantooth, Denita Hadziabdic, Sarah Boggess, Mark Windham, Stephen Miller, Guohong Cai, Joseph Spatafora, Ning Zhang, Meg Staton, Bonnie Ownley, Robert Trigiano

**Affiliations:** 1 Department of Entomology and Plant Pathology, University of Tennessee, Knoxville, Tennessee, United States of America; 2 Department of Plant Biology and Pathology, Rutgers University, New Brunswick, New Jersey, United States of America; 3 APHIS PPQ, Linden, New Jersey, United States of America; 4 Crop Production and Pest Control Research Unit, Agricultural Research Service, United States Department of Agriculture, West Lafayette, Indiana, United States of America; 5 Department of Botany and Plant Pathology, Purdue University, West Lafayette, Indiana, United States of America; 6 Department of Botany and Plant Pathology, Oregon State University, Corvallis, Oregon, United States of America; Natural Resources Canada, CANADA

## Abstract

*Cornus florida* (flowering dogwood) and *C*. *nuttallii* (Pacific dogwood) are North American native tree species that belong to the big-bracted group of dogwoods. *Cornus* species are highly valued for their ornamental characteristics, and have fruits that contain high fat content for animals. Also, they are an important understory tree in natural forests. Dogwood anthracnose, caused by *Discula destructiva*, was observed in the late 1970s on the east and west coasts of the United States and by 1991 had quickly spread throughout most of the native ranges of *C*. *florida* and *C*. *nuttalli*. We investigated the genetic diversity and population structure of 93 *D*. *destructiva* isolates using 47 microsatellite loci developed from the sequenced genome of the type strain of *D*. *destructiva*. Clone-corrected data indicated low genetic diversity and the presence of four genetic clusters that corresponded to two major geographic areas, the eastern United States and the Pacific Northwest, and to the two collection time periods when the isolates were collected (pre- and post-1993). Linkage disequilibrium was present in five out of six subpopulations, suggesting that the fungus only reproduced asexually. Evidence of population bottlenecks was indicated across four identified genetic clusters, and was probably the result of the limited number of founding individuals on both coasts. These results support the hypothesis that *D*. *destructiva* is an exotic pathogen with independent introductions on the east and west coasts of North America. We also tested the cross-amplification of these microsatellite primers to other *Discula* species. Genomic DNA from 17 isolates of four other *Discula* species and two isolates of *Juglanconis* species (formerly *Melanconis* species) were amplified by 17 of 47 primer pairs. These primers may be useful for investigating the genetic diversity and population structure of these *Discula* species.

## Introduction

*Cornus florida* L. (flowering dogwood) and *C*. *nuttallii* Aud. (Pacific dogwood) are tree species native to North America and classified in the big-bracted group of dogwoods [1]. *Cornus florida* is widespread throughout eastern North America from southeastern Ontario, to Canada in the north, to northern Florida in the south, and as far west as eastern Texas [2], whereas *C*. *nuttallii* is endemic to western North America and ranges from southwestern British Columbia to northern California [3] with small disjunct populations in northern Idaho and southern California [4]. *Cornus florida* is a major component of the understory in eastern hardwood forests [3] and provides food for numerous species of birds and mammals [1, 5]. The trees are highly prized as ornamentals because of their attractive springtime bract display, red or yellow berries, and attractive fall foliage. *Cornus florida* nursery sales in the U.S.A. exceeded $27 million in 2014 with more than $6.5 million from Tennessee nurseries [6].

Most foliar diseases of *C*. *florida* cause only cosmetic damage requiring no intervention. However, powdery mildew and dogwood anthracnose can be very problematic for commercial production and cause considerable harm to individual trees as well as entire populations. Powdery mildew, a fungal disease caused by *Erysiphe pulchra* [7], typically disfigures leaves, stunts the growth of plants, and reduces flower and fruit production [8, 9]. Although powdery mildew can be damaging to dogwoods, the impact is not as severe as dogwood anthracnose, which can kill trees in landscapes and native forests typically in one to three years. Dogwood anthracnose, caused by *Discula destructiva*, is a very devastating disease that was first observed on *C*. *nuttallii* during 1976 in Washington State [10], whereas the first documented incidence of the disease in the eastern U.S.A. on *C*. *florida* was in 1977 in New York [11]. Thousands of *C*. *florida* trees within New York and Connecticut were either killed or extensively damaged by what was then considered a new “mysterious” disease [12].

The disease spread across the natural western range of *C*. *nuttallii* [10] including a disjunct population in Idaho [13, 14]. In the eastern U.S.A., the area affected by dogwood anthracnose expanded rapidly throughout the northeast and southward along the spine of the Appalachian Mountains. By 1987, diseased trees were found in natural areas of Georgia [15]. In 1988, it was determined that both *C*. *nuttallii* in the west and *C*. *florida* in the east were being affected by the same disease [16]. *Discula destructiva* has several known modes of dispersal. Localized dissemination has been demonstrated by splashing water in the form of rain or overhead irrigation [17]. Insects that encounter conidia may also play an important role in local and regional dissemination of the pathogen [18]. Animals, and especially migratory birds, are probably involved in long-distance spread of the pathogen by transporting infected fruits and seeds to new locations [17]. Humans have contributed to the spread of dogwood anthracnose by moving infected plants from one country to another. Infected *C*. *florida* trees were exported from the U.S.A. to the United Kingdom in 1995 [19], and the disease has since been found in Germany [20], Italy [21], and Switzerland [22].

The first signs and symptoms of the disease typically are found on leaves and young shoots, and infection may progress into twigs, which die back. The hyphae may infect the main trunk through progression from the base of infected epicormic shoots to cause sunken, necrotic annual cankers; the lesions primarily affect phloem tissues [23].Canker formation on the trunk is especially problematic as individual cankers often coalesce and girdle the tree. Epicormic buds along the lower portion of the trunk become activated by the loss of apical dominance caused by the death of apical meristems on the leader or other infected branches [23]. Dogwood anthracnose can affect any size and age of flowering dogwood tree [24] and the impacts of the disease can be devastating. High mortality rates have been documented in Catoctin Mountain Park, Maryland [25]. A 1984 disease impact survey revealed that 33% of *C*. *florida* trees had been killed by dogwood anthracnose, and only 3% of trees were free of symptoms [26]. In a follow-up survey in 1994, 94% of dogwood trees in the park had died [27]. Surveys conducted in the Great Smoky Mountain National Park indicated that dogwood anthracnose had killed 25% of *C*. *florida* trees by 1992 and 75% just two years later [18]. Japanese or Chinese dogwood (*C*. *kousa* Hance), which is non-native to North America, displays some resistance to dogwood anthracnose [11]. Infected *C*. *kousa* trees may exhibit small leaf spots, but shoot dieback is not as severe, and tree mortality rarely occurs [28, 29].

The causal agent of dogwood anthracnose was initially thought to be a *Gloeosporium* species in the west [10] and *Colletotricum gleosporioides* in the east [12]. Fulfillment of Koch’s postulates revealed *Discula* species as the cause of the disease, which was later characterized as a new species, *D*. *destructiva* [25]. However, not all the fungi isolated from diseased dogwood tissue fit the description of *D*. *destructiva*, and hence two types of *Discula* species associated with dogwood anthracnose were designated as *Discula* Type I (*D*. *destructiva*) and Type II, an unnamed species of *Discula* [16, 30]. Besides some morphological differences between the two types of fungi, all *D*. *destructiva* isolates produced phenol oxidases that oxidized gallic acid and other phenolic substrates, whereas none of the *Discula* type II species could not oxidize any of these substrates [30]. *Discula destructiva* is a haploid, ascomycetous fungus in the order Diaporthales, and the Gnomoniaceae [31, 32]. Some other *Discula* species are anamorphs of *Apiognomonia*, but a teleomorph has not been observed for *D*. *destructiva* on diseased materials or in the laboratory [25]. Conservation of the anamorphic genus *Discula* for dogwood anthracnose has been argued because of the lack of a sexual stage [33].

*Discula destructiva* may be either an indigenous species that had gone unnoticed until the late 1970s or an introduced pathogen [26]. The former explanation is unlikely because of the sudden appearance, dramatic effects, and apparent spread of the pathogen. In addition, the lack of resistance in native populations, provides additional evidence and support that the latter account is more plausible. Phylogenetic analyses of the internally transcribed spacer region (ITS) indicated that *D*. *destructiva* did not arise from a native population [34]. DNA amplification fingerprinting (DAF) [35] reported very little genetic variability among eastern and western *D*. *destructiva* isolates, further supporting the exotic invader hypothesis. The DAF method generates an abundance of data, but amplicons cannot be linked to specific genomic sites or loci. Furthermore, a single band in a gel may contain multiple amplicons, which may or may not be present among all the isolates. Analysis of arbitrary signatures from amplification profiles (ASAP) [36] detected low genetic diversity among isolates, and the inferred population structure supported the recent and separate introductions on the east and west coasts of North America. ASAP dissects DAF products by finding complimentary sites with hairpin primers within the amplicons and is capable of finding differences more related to sequences of amplicons instead of primer sites [37]. Banding profiles of double stranded RNA (dsRNA) indicated that eastern and western isolates of *D*. *destructiva* likely have separate origins [38, 39]. Amplified fragment length polymorphisms (AFLPs) [40] also revealed two distinct groups representing isolates from the east and west coasts of North America, further supporting the hypothesis of separate introductions. This study also found a higher level of genetic diversity in isolates collected in VA, PA, MD and NJ compared to isolates collected in areas further north and south where the disease was reported later, thus supporting the hypothesis that New York City may be the disease epicenter in the eastern U.S.A. Lastly, *D*. *destructiva* was identified by qPCR from *Cornus* specimens collected before the first report of the dogwood anthracnose in North America from China and Japan, the hypothesized center of origin, where it may exist as an endophyte [41].

To elucidate the pathogen origin and spread in the U.S.A., we investigated genetic diversity and spatial structure of *D*. *destructiva* using microsatellite loci. Microsatellite loci or simple sequence repeats (SSRs) are repeating units of nucleotides that occur throughout the genome of eukaryotic organisms that can be used as genetic markers [42]. Compared to the arbitrary primer methods employed in previous studies, microsatellite loci can enable a more reliable interpretation of the population diversity and structure of *D*. *destructiva*. They facilitated greater detection of genetic diversity by amplifying discrete or defined loci compared to arbitrary primers [43], and therefore, will allow evaluation of the same genomic loci among samples or isolates. Therefore, the specific objectives of our study were the following: 1) to develop and test microsatellite loci for population studies of *D*. *destructiva*; 2) to identify spatial structure and genetic diversity of *D*. *destructiva* isolates collected across North America from different time periods; and 3) to test the cross-amplification of these microsatellite loci in other *Discula* species.

## Materials and methods

### *Discula destructiva* isolates

Ninety-three isolates of *D*. *destructiva* collected from 1989–2000 across eastern and western North America were used in this study. Nineteen isolates of *D*. *destructiva* were obtained from a collection at Rutgers University (New Brunswick, New Jersey, U.S.A.) and fifty isolates were supplied from a collection at the University of Tennessee (UTK; Knoxville, Tennessee, U.S.A.). Genomic DNA of 24 other *D*. *destructiva* isolates, as well as genomic DNA from isolates of five other *Discula* and *Juglanconis* species were available from the UTK collection (Table 1). Stock cultures of *D*. *destructiva* isolates were maintained on potato dextrose agar (PDA; Sigma-Aldrich, St. Louis, Missouri, U.S.A.) for 10–14 days at room temperature (ca. 20°C) before being used to inoculate medium to grow mycelia for DNA isolation. Stock cultures were transferred to fresh medium every two months. Small pieces (ca. 5 mm^2^) of mycelium were harvested from the periphery of 14-day-old fungal colonies and used to inoculate sterilized cellophane (Bio-Rad Laboratories, Hercules, California, U.S.A.) [44] placed on PDA contained in 60-mm Petri dishes. Cultures were grown at room temperature for 14 days. Fungal mycelia were flash frozen in liquid nitrogen and ground into a fine powder with a sterile mortar and pestle. Genomic DNA was extracted using the Qiagen DNeasy Plant Mini Kit (Qiagen, Valencia, California, U.S.A.) following the manufacturer’s protocol with the following modifications: 1.5% of the total volume of polyvinylpyrrolidone (PVP) was added to the lysis buffer (AP1), and the initial incubation time was increased to 20 min. Genomic DNA was quantified using the NanoDrop ND-1000 Ultraviolet-Vis Spectrophotometer (NanoDrop Technologies, Wilmington, Delaware, U.S.A.) and stored at -20°C until needed for amplification.

**Table 1:** Isolates of *Discula destructiva* and *Discula* species used in this study.

*Discula* species	Culture No.	Collection^a^	Host species	Location^b^	Location/Year^c^
*D*. *destructiva*	AL151	UTK	*C*. *florida*	AL	S/1992
	AS1	UTK	*C*. *florida*	TN	S/2000
	AS11	UTK	*C*. *florida*	TN	S/2000
	AS111	UTK	*C*. *florida*	TN	S/2000
	AS12	UTK	*C*. *florida*	TN	S/2000
	AS14	UTK	*C*. *florida*	TN	S/2000
	AS15a	UTK	*C*. *florida*	TN	S/2000
	AS15b	UTK	*C*. *florida*	TN	S/2000
	AS18a	UTK	*C*. *florida*	TN	S/2000
	AS18b	UTK	*C*. *florida*	TN	S/2000
	AS22	UTK	*C*. *florida*	TN	S/2000
	AS32	UTK	*C*. *florida*	TN	S/2000
	AS37	UTK	*C*. *florida*	TN	S/2000
	AS39	UTK	*C*. *florida*	TN	S/2000
	AS4	UTK	*C*. *florida*	AL	S/2000
	AS51	UTK	*C*. *florida*	TN	S/2000
	AS58	UTK	*C*. *florida*	TN	S/2000
	AS85	UTK	*C*. *florida*	TN	S/2000
	AT4	UTK	*C*. *florida*	VA	N/Pre-1993
	BC	UTK	*C*. *nuttallii*	BC	W/Pre-1993
	BT4	UTK	*C*. *florida*	TN	S/1991
	CORNU2	UTK	*C*. *nuttallii*	OR	W/Pre-1993
	E3c	UTK	*C*. *florida*	PA	N/1990
	E11	UTK	*C*. *florida*	TN	S/1991
	E87	UTK	*C*. *florida*	TN	S/1993
	E90	UTK	*C*. *florida*	PA	N/1990
	E114	UTK	*C*. *florida*	TN	S/1993
	E118	UTK	*C*. *florida*	TN	S/1989
	E124	UTK	*C*. *florida*	PA	N/1990
	E128	UTK	*C*. *florida*	PA	N/1990
	E145	UTK	*C*. *florida*	TN	S/1991
	E170	UTK	*C*. *florida*	TN	S/1991
	GA1	UTK	*C*. *florida*	GA	S/1989
	GR325	UTK	*C*. *florida*	NY	N/Pre-1993
	MDRR1	RU	*C*. *florida*	MD	N/Pre-1993
	M10	UTK	*C*. *nuttallii*	OR	W/Pre-1993
	M10b	UTK	*C*. *nuttallii*	OR	W/Pre-1993
	M18a	UTK	*C*. *nuttallii*	OR	W/Pre-1993
	M28e	UTK	*C*. *nuttallii*	OR	W/Pre-1993
	MA18	UTK	*C*. *kousa*	MA	N/1989
	MAF2	RU	*C*. *florida*	MA	N/1989
	MAK2	RU	*C*. *kousa*	MA	N/1989
	MAK5	RU	*C*. *kousa*	MA	N/1989
	MDDR9	RU	*C*. *florida*	MD	N/Pre-1993
	MD1_utk	UTK	*C*. *florida*	MD	N/1990
	MD1_ru	RU	*C*. *florida*	MD	N/1999
	MDRR1	RU	*C*. *florida*	MD	N/1999
	MDRR2	RU	*C*. *florida*	MD	N/1999
	MDRR4	RU	*C*. *florida*	MD	N/1999
	NJ135	UTK	*C*. *florida*	NJ	N/Pre-1993
	PA5	RU	*C*. *florida*	PA	N/1989
	PA129	UTK	*C*. *florida*	PA	N/Pre-1993
	PA-031013-3	RU	*C*. *florida*	PA	N/Pre-1993
	PA-031013-5	RU	*C*. *florida*	PA	N/Pre-1993
	PA-0308203	RU	*C*. *florida*	PA	N/Pre-1993
	PA-0308205	RU	*C*. *florida*	PA	N/Pre-1993
	PA-5	RU	*C*. *florida*	PA	N/1989
	RI10	UTK	*C*. *florida*	TN	S/1991
	RI16	UTK	*C*. *florida*	TN	S/1991
	RI18	UTK	*C*. *florida*	TN	S/1991
	RI20	UTK	*C*. *florida*	TN	S/1991
	RI21	UTK	*C*. *florida*	TN	S/1991
	RI5	UTK	*C*. *florida*	TN	S/1991
	RI9	UTK	*C*. *florida*	TN	S/1991
	SC101	UTK	*C*. *florida*	SC	S/Pre-1993
	SC106	UTK	*C*. *florida*	SC	S/1990
	SG11	UTK	*C*. *florida*	TN	S/1991
	SG15	UTK	*C*. *florida*	TN	S/1991
	SG21	UTK	*C*. *florida*	TN	S/1991
	SG23	UTK	*C*. *florida*	TN	S/1991
	SG6	UTK	*C*. *florida*	TN	S/1991
	SG8	UTK	*C*. *florida*	TN	S/Pre-1993
	Shadow1	UTK	*C*. *florida*	TN	S/1991
	Shadow2	UTK	*C*. *florida*	TN	S/1991
	Sig.Mtn.1	UTK	*C*. *florida*	TN	S/1991
	TN1	UTK	*C*. *florida*	TN	S/1989
	TN12	UTK	*C*. *florida*	TN	S/Pre-1993
	TN14	UTK	*C*. *florida*	TN	S/Pre-1993
	TN15	UTK	*C*. *florida*	TN	S/Pre-1993
	TN16	UTK	*C*. *florida*	TN	S/Pre-1993
	TN22	UTK	*C*. *florida*	TN	S/Pre-1993
	TN24	UTK	*C*. *florida*	TN	S/Pre-1993
	TN8	UTK	*C*. *florida*	TN	S/1989
	Univ. of South2	UTK	*C*. *florida*	TN	S/1991
	VA161	UTK	*C*. *florida*	VA	N/Pre-1993
	W64	UTK	*C*. *nuttallii*	OR	W/1994
	W94	UTK	*C*. *nuttallii*	WA	W/1994
	W133	UTK	*C*. *nuttallii*	CA	W/Pre-1993
	W145	UTK	*C*. *nuttallii*	CA	W/1994
	W162	UTK	*C*. *nuttallii*	CA	W/1994
	WAP2	RU	*C*. *nuttallii*	WA	W/1990
	WAP272	RU	*C*. *nuttallii*	WA	W/1990
	WAP31	RU	*C*. *nuttallii*	WA	W/1989
	WAP51	RU	*C*. *nuttallii*	WA	W/Pre-1993
*D*. *umbrinella*	67	OP	*Quercus robur*	Switzerland	Pre-1993
	115	OP	*Castanea* (Ct.) *sativa*	Switzerland	Pre-1993
	116	OP	*Ct*. *sativa*	Switzerland	Pre-1993
	221	OP	*Fagus sylvatica*	Switzerland	Pre-1993
	319	OP	*Q*. *robur*	Switzerland	Pre-1993
	324	OP	*Q*. *robur*	Switzerland	Pre-1993
	416	OP	*Ct*. *sativa*	Switzerland	Pre-1993
	427	OP	*Ct*. *sativa*	Switzerland	Pre-1993
	510	OP	*F*. *sylvatica*	Switzerland	Pre-1993
	518	OP	*F*. *sylvatica*	Switzerland	Pre-1993
	611	OP	*Q*. *robur*	Switzerland	Pre-1993
	617	OP	*Q*. *robur*	Switzerland	Pre-1993
	P4	OP	*F*. *sylvatica*	Poland	Pre-1993
	LT135	OP	*F*. *sylvatica*	France	Pre-1993
	LTO68	OP	*Q*. *robur*	Great Britain	Pre-1993
*D*. *quercina*	DQB	OP	*Q*. *garryana*	Switzerland	Pre-1993
	LOMtA	UTK	*C*. *florida*	TN	1992
*D*. *campestris*	DCAMP	GS	*Acer saccharum*	WI	1989
*D*. *fraxinea*	89-32-2	GS	*Fraxinus americana*	PA	1993
*Discula-*like fungus (Type II)^d^					
	VA17B	UTK	*C*. *florida*	VA	1991
	NC2	UTK	*C*. *florida*	NC	1991

^a^ Collections from University of Tennessee, Knoxville (UTK); Rutgers University (RU); and received from Dr. Orlando Petrini (OP) Microbiology Institute and Department of Forest and Wood Sciences, Federal Institute of Technology, ETH-Zentrum, Zurich, Switzerland (current address: POLE Pharma Consulting, Ticino, Switzerland), and Dr. Glen Stanosz (GS), University of Wisconsin, Madison.

^b^ Locations use United States postal state designations unless otherwise noted. BC—British Columbia.

^c^ Location: S = South and N = North in Eastern United States, and W = Western North America. If year of isolation is unknown, then it is labeled as pre-1993.

^d^ Identified as a species of *Juglanconis* species (formerly *Melanconis* species).

### Verification of *Discula destructiva*

All living isolates from the UTK and RU collections were verified as *D*. *destructiva* by amplifying the ITS region of fungal ribosomal DNA using the polymerase chain reaction (PCR). Each 30 μl reaction contained the following: 6 ng genomic DNA template, 1.5 μl of 5 μM each of ITS1 (5’—TCCGTAGGTGAACCTGCGG—3’) and ITS4 (5’—TCCTCCGCTTATTGATATGC—3’)[45], 3 μl of 25 mM MgCl_2_, 3 μl of 10X PCR Gold Buffer (Applied Biosystems, Austin, Texas, U.S.A.), 1.5 μl dimethyl sulfoxide (DMSO; Fisher Scientific, Pittsburgh, Pennsylvania, U.S.A.), 0.24 μl of 5 U/μl AmpliTaq Gold® DNA polymerase (Applied Biosystems), 3 μl of 2.5 mM deoxynucleoside triphosphate (dNTPs), and 10.26 μl sterile distilled water. The thermal cycler (Eppendorf AG, Hamburg, Germany) was programmed with initial denaturation of 95°C for 3 min followed by 35 cycles of 95°C for 1 min, 50°C for 30 s, 72°C for 80 s, and a final extension at 72°C for 10 min. Five μl of ITS PCR products were visualized with ethidium bromide on 2% agarose gels using a 2000 Gel Documentation System (BIO-RAD). PCR amplicons were purified using Qiagen QIAquick purification kit (Qiagen, Germany) according to the manufacturer’s instructions. The purified DNA was quantified using NanoDrop ND-1000 Ultraviolet-Vis Spectrophotometer and sequenced at the UTK Genomics Core (http://mbrf.utk.edu/). Fungal isolates were confirmed as *D*. *destructiva* by comparing the ITS sequences to known sequences in the GenBank database using the Basic Local Alignment Search Tool (BLAST) available from the National Center for Biotechnology Information (NCBI) website (http://www.ncbi.nlm.nih.gov/). The ITS sequences of the 66 isolates in live culture were aligned using BIOEDIT v7.2.5 (http://www.mbio.ncsu.edu/bioedit/bioedit.html). The 24 pre-1993 *D*. *destructiva* isolates (DNA only) and 21 *Discula* species were confirmed previously using the same procedure except that the primers ITS1 and IST2 [45] were used (unpublished data).

### Developing and identifying polymorphic microsatellite loci

*Discula destructiva* type isolate MD235 was used for genome sequencing. A paired-end DNA library (Illumina) was made according to manufacturer’s instructions and sequenced to produce 74.9 million 100 bp x 2 read pairs. The reads were filtered by requiring a minimal quality score of 20 in at least 70% of the bases. Filtered reads were assembled using SOAPdenovo2 [46] with kmer = 53. Microsatellite loci were identified with a custom Perl script (https://github.com/statonlab/disculaSSRs). Di-, tri-, and tetra- nucleotide perfect repeats were only reported if they met the following criteria: di- nucleotide motifs with 8–40 repeats, tri-nucleotide motifs with 7–30 repeats, and tetra-nucleotide motifs with 6–20 copies. Sequences were masked for low complexity regions with DUSTMASKER [47] and primers designed for repeats using PRIMER3 v2.3.6 [48]. The following parameters were altered from the Primer3 default settings: maximum primer size was 25; primer product size ranged between 100 and 200 base pairs [49]; primer minimum and maximum annealing temperature was 55°C and 65°C respectively, primer minimum GC percentage was between 40 and 60; the maximum allowable length of a mononucleotide repeat (mx poly-x) was set to 3; and primer GC clamp was set to 2.

Primer pairs for 50 microsatellite loci (Table 2) with annealing temperatures between 59–63°C were selected and then manufactured by Integrated DNA Technologies (Coralville, Iowa, U.S.A.). Initially four isolates were randomly chosen from the UTK collection and used to screen informative loci. A 10 μl amplification reaction contained the following: 1 μl of 0.25–2 ng/μl genomic DNA template, 1 μl of 2.5 μM of each primer pair, 1 μl of 25 mM MgCl_2_, 1 μl of 10X PCR Gold buffer (Applied Biosystems), 0.5 μl DMSO, 0.1 μl of 5 U**/**μl AmpliTaq Gold® DNA polymerase, 1 μl (2.5 mM) dNTPs, and 4.4 μl sterile, distilled water. Reactions were completed in an automatic thermal cycler under the following conditions: initial denaturation at 96°C for 4 min, followed by 35 cycles of denaturation at 95°C for 30 s, annealing at 56°C for 30 s, extension at 72°C for 30 s, and a final extension at 72°C for 5 min.

**Table 2:** Forty-seven microsatellite loci used to assess genetic diversity of *Discula destructiva* isolates and cross-amplification to other *Discula* species.

GenBank Accession No.	Locus	Forward and Reverse Primers (5'-3')	Repeat Motif	No. of Alleles	Allele Range	Gene Diversity
KX953766	DD01	F:GGAAGTACTCAGCCTCAGCC R:GAGGTCTGGTGGTGTTGAGG	(CAT)_17_	3	174–268	0.18
KX953767	DD02	F:GTTGAGTCGAGTGGTGGAGG R:GATTGCCCTCACCCTCATCC	(GTG)_8_	1	162	0.00
KX953768	DD03	F:TGCTTGTGTTGTCCGAATGC R:GGCACATAGACGCGCTATCG	(TGC)_11_	2	140–144	0.03
KX953769	DD04	F:AATAGGTGCTCAGTTGGCGG R:AACGTCGACAGCCTTCTACG	(TGC)_8_	1	150	0.00
KX953770	DD05	F:TTAAAGGATCGCATCGTGGC R:AACTCAAATGCAGCAAGCCG	(TGC)_13_	4	115–170	0.08
KX953771	DD06	F:CATGATCATCTTCGCCGTCG R:CAGGTTGAGCGCATGATGC	(CCA)_8_	1	197	0.00
KX953772	DD07	F:ATCCCGGCTATGCTCTTTCG R:AGGAGGATGGTGGCAATAGC	(TTG)_8_	2	185–188	0.05
KX953773	DD08	F:CTAGCTCAGGATCAGCGACG R:AGGAGTACGAACGTGAACGC	(GAG)_7_	2	136–139	0.02
KX953774	DD09	F:CCTGGCACTCTCTCGATTTCC R:ATGATCATGACCGGTCTCGC	(CCG)_8_	1	161	0.00
KX953775	DD10	F:TCCTCATCCATTCGTTGCGG R:GCTCAAAGTACATCAACAGACTCC	(TGT)_7_	2	149–154	0.03
KX953776	DD11	F:CATCCTCGACTCTGATGGCC R:CGGTGCCATGACATAACTGC	(CCA)_7_	1	102	0.00
KX953777	DD12	F:ATCAAGAGTCCACCCACTCG R:TGGACAGATTGGGTGAGTGC	(GGT)_7_	2	161–164	0.46
KX953778	DD13	F:CGACCAGAACCATGACCACC R:CCTTCTCTGGTCTACCTGGC	(CCT)_9_	3	178–186	0.33
KX953779	DD14	F:ACAGTTGAGGTCGTAAGCGG R:AGCAGCTCCAGAAGAACACC	(TGG)_8_	2	191–196	0.43
KX953780	DD15	F:CACTCAACAACAGCGACACC R:TGCGGAGTGTCATATGAGGC	(GCA)_9_	3	129–153	0.05
KX953781	DD16	F:AAACCCAAACGACAATGCCG R:ATTAGCTGGGCCGCTGTTGG	(CAC)_8_	2	192–197	0.10
KX953782	DD17	F:TCTTGCGCGGTAATGTCTCC R:ACCTGTAAACAAGATGAACGCC	(CTA)_16_	4	162–182	0.08
KX953783	DD18	F:GTTGCGCCGTTTGTAGTGC R:CTTCGAATCGCACGTCTTCG	(CAG)_15_	2	164–176	0.03
KX953784	DD19	F:GTGCTGCTGTTGACTTGTGG R:GTAGCCTCTCCGAATCAGCC	(TGC)_7_	2	163–166	0.21
KX953785	DD20	F:TTTGCTGAAGGAGGTACGGC R:GCATCAGCATCAGCATCAGC	(TGG)_11_	2	146–149	0.24
KX953786	DD22	F:CTTGGTGGGCTGTTTGTGC R:AGACGACAACACCAGCATCG	(GGT)_7_	2	192–197	0.26
KX953787	DD23	F:AGAAGCTGTCAAGAGGCACC R:AAGCGGAGTTCTGAGACAGG	(ACC)_12_	4	184–200	0.33
KX953788	DD24	F:CAGCTTACAACAGGTCAAGGC R:GTAGAGAGAGGATCCTGCGG	(AGC)_7_	5	176–193	0.27
KX953789	DD25	F:TCAAACTCAGACTCGCTGCC R:GAACCAGGTCTCCAAGCAGC	(GAG)_7_	3	179–185	0.17
KX953790	DD26	F:AGTCGCTCTTCTCAACGAGG R:CTGAATTGAGCAGCGGCAGC	(CAG)_9_	3	179–189	0.25
KX953791	DD27	F:GTCTCTGACTGAACCTCCGC R:GAAGGACGATGCTCTCTCGG	(ATT)_9_	2	150–156	0.25
KX953792	DD28	F:AATAAACAGCACACACCGCC R:ATTGTTTGGTTGATGGCGGG	(AGA)_9_	3	176–185	0.35
KX953793	DD29	F:CGATGCCAGCGGTTTAAACG R:GCAGACGCTCATGATTTCCC	(AGC)_7_	2	123–126	0.30
KX953794	DD30	G F:CATTGTACTCAGAGGCCCG R:CAGATCAACAACTGCCAGGC	(TGC)_7_	2	199–203	0.22
KX953795	DD31	G F:TGAAGACGGTTGACTGTGC R:CATTGAGAATCTGCTGCACCG	(AG)_10_	2	163–167	0.24
KX953796	DD32	F:TCACATGAAGGAGACGAGCG R:CTCTTTCACCACCTCCTCCC	(AG)_9_	2	157–160	0.12
KX953797	DD33	F:CCAAGGGTAGATGGTCAAGCC R:AGGAAGCAAAGGGAGGATTGG	(TC)_11_	2	184–187	0.20
KX953798	DD34	F:GAAAGACACTGCACAAGCCG R:CCTGGAGAGCAGAACAGTGG	(GT)_9_	2	197–200	0.23
KX953799	DD35	F:ATACCAGCTTCAGCCCATGG R:CTCTGTGTTGGTGTATGCGC	(AC)_11_	2	193–196	0.01
KX953800	DD36	F:TACACTCACCAAGCATCGCC R:AGGCCTGGTAAGCAAGTTGG	(TC)_12_	2	195–200	0.41
KX953801	DD37	F:GTCACCAGGAATAGGACCGC R:GGATGACCTGGGACTCTTGC	(AC)_15_	2	160–163	0.21
KX953802	DD39	F:TTTGATCATTCTCGGCCGCC R:AGAGTCATCGCATGGTTCCG	(CT)_9_	1	127	0.00
KX953803	DD40	F:AGGCTTGCCTAATCGAAGCG R:ACAAAGGAGCTGCTTGTAGC	(TA)_8_	2	159–164	0.25
KX953804	DD41	F:AGACCTTGATCGGAGACAGC R:CATGATTGGCTTTCGGCTGG	(TC)_10_	1	136	0.00
KX953805	DD42	F:AATGGATTCCTGTCGCTCGG R:GAGCGAGCGAGTGCATATCC	(TA)_18_	3	146–152	0.15
KX953806	DD43	F:TTCCACCATGCAATGCAACC R:GAAGAGCCCGAGTTGTTTGC	(AC)_12_	2	108–113	0.29
KX953807	DD44	F:CGAACGTTGCTGTATGTGCC R:AGTGTTCCGAGTTGTACCGG	(AG)_10_	2	136–141	0.32
KX953808	DD45	F:TCCTAGGCACTTTGATGGCG R:CTCGGTCAACGGCATAGTGG	(CA)_8_	3	140–148	0.33
KX953809	DD46	F:CTTGTTGGGTTGCGAAACGG R:GGCCGACTGTGATATCACCG	(TG)_10_	2	188–193	0.31
KX953810	DD47	F:AGGTATGCTCACTATGGCGCT R:TGATGGTGTTCTCGGTCCG	(GA)_13_	2	167–172	0.31
KX953811	DD49	F:ATGTCAAGGTGAGTCAGGCG R:CAATGGCTTCCACCAATGGC	(TC)_11_	3	154–160	0.18
KX953812	DD50	F:ATGGCTGTCATGCACAATCC R:TGTTGTCGTGGATGGATGGG	(TA)_8_	2	175–178	0.21

PCR products were analyzed on a QIAxcel Capillary Electrophoresis System (QIAGEN, Valencia, California, U.S.A.) using an internal 25-bp size standard to provide raw allele length data. Forty-seven primer pairs (Table 2) that produced unambiguous products were selected for the study and used to amplify DNA of the 93 *D*. *destructiva* isolates and other *Discula s*pecies isolates. Reactions that did not produce a product were repeated at least once before assuming that the loci of these isolates were either a null allele or considered missing data for the analyses.

### Population diversity

FLEXIBIN v2 [50] was used to automatically bin raw allele length data into allelic classes; binned data were used in all the following analyses. Identical multilocus *D*. *destructiva* haplotypes were identified using POPPR v2.1.1 [51] a package for R v3.3.1 [52]. All isolates (n = 93) used in this study were grouped into the following six populations based on geographic location in North America and time of collection (pre- and post-1993): pre-1993 North, pre-1993 South, pre-1993 West, post-1993 North, post-1993 South, and post-1993 West. Pre- and post- 1993 North were defined as isolates from northern Virginia, Maryland, Massachusetts, New Jersey, New York, and Pennsylvania, U.S. Due to its close proximity and limited number of isolates (n = 2), we placed the VA isolates in the northern group, although VA is historically considered a southern state. We defined pre- and post- 1993 South as isolates from southern states including Alabama, Georgia, South Carolina, and Tennessee. North and South are also generally referred to as “eastern isolates”. Pre-and post-1993 West were defined as isolates originating from California, Oregon, and Washington U.S., and British Columbia, Canada. All six subpopulations were used for subsequent analyses. The data set was clone corrected and 80 haploid individuals with 69 unique multilocus genotypes (MLG) across six identified subpopulations were used for subsequent analyses. Clone-correction is a common procedure for clonally reproducing organisms to ensure that the allele frequencies are not overestimated due to over-representation of certain haplotypes, and that each MLG is represented only once across six subpopulations [53, 54].

The program GenAlEx v6.5 [55, 56] and POPPR were used to calculate measures of genetic diversity across all loci and collection sites. Pairwise population differentiation and gene flow among *D*. *destructiva* subpopulations were assessed using ARLEQUIN v3.5.2 [57] where non-significant values were an indicator of a recent/ historical gene flow between particular subpopulations. The standardized index of association (${\bar{r}}_{d}$) [58] was estimated using 10,000 permutations, and the null hypothesis ${\bar{r}}_{d}$ = 0 (linkage equilibrium) was tested in POPPR. This unbiased index of association detects signatures of multilocus linkage and potential clonal reproduction within populations. The standardized index of association ${\bar{r}}_{d}$ correspond to the index of association I_A,_ but is considered less biased because it is independent of the number of loci used in the study. In addition, the value of ${\bar{r}}_{d}$ is expected to be zero in panmictic populations, whereas clonally reproducing organisms should have values significantly greater than zero [59].

### Population structure and genetic differentiation

Genetic clustering and population structure were analyzed using the program STRUCTURE v2.3.4 [60] with a Bayesian Monte Carlo Markov Chain (MCMC) clustering method. The burn-in period was 500,000 with 500,000 MCMC repetitions using 30 iterations of K = 1–10. STRUCTURE HARVESTER web v0.6.94 [61] was utilized to estimate the optimum value of K (genetic clusters) using the method of Evanno et al. [62] and visualized using R package POPHELPER [63]. Genetic differentiation analysis of molecular variance (AMOVA) was performed with ARLEQUIN v3.5.2 [57]. The five variance partitions used for the AMOVA included the following: all subpopulations grouped as one hierarchical group, partitioned based on two time periods (pre-1993 and post-1993), partitioned based on two geographic regions (eastern and western North America), partitioned based on different host species–*C*. *florida*, *C*. *nuttallii*, and *C*. *kousa*, and lastly, partitioned based on STRUCTURE findings. For AMOVA analyses, the following variance components were calculated: i) among groups (*F*ct), ii) among subpopulations within groups (*F*sc), and (iii) among subpopulations (within individuals) (*F*st). All AMOVA analyses were computed using 10,000 permutations at *P*<0.05 significance level.

Principal coordinate analysis (PCoA) implemented in GenAlEx, and dendrograms created in POPPR using Nei’s genetic distance [64] and Bruvo’s method [65], were used to visualize the genetic relationship among the clusters. Bruvo’s distance was utilized to construct and visualize Unweighted Pair Group Method using Arithmetic Averages (UPGMA) algorithms using 1,000 bootstraps with support values greater than 50%. Unlike Nei's genetic distance, Bruvo’s distance is based on genetic distance between individuals, not populations [51].

### Historical demography

The program BOTTLENECK v1.2.02 [66] was used to evaluate if a recent population bottleneck or expansion had occurred, and groups were based on the number of genetic clusters as defined by STRUCTURE. The data was coded as a diploid, as described in Tsui et al. [67] for analysis in BOTTLENECK, and both Sign and Wilcoxon significance tests were utilized to determine if the 47 loci used in this study remained in mutation-drift equilibrium. In populations where the effective size remains constant, the probability of any given locus to display heterozygosity excess or a heterozygosity deficit are equally plausible. The following three mutation models were used: infinite allele model (I.A.A.), stepwise mutation model (S.M.M.), and two phase model (T.P.M.) using default settings.

## Results

### Verification of *Discula destructiva*

A single band of ~550 bp was amplified using ITS1 and ITS4 primers for the previously uncharacterized 69 isolates of *D*. *destructiva* from RU and UTK. The amplicon sequences were matched to *D*. *destructiva* (the most common Accession Number was AF429741.1; all queries had an E-value = 0; 99–100% identity). The DNA of pre-1993 isolates from the UTK collection was previously confirmed as *D*. *destructiva* in a similar manner except ITS1 and ITS2 [45] were used. Sequencing of the ITS region of *Discula*-like or Type II isolates with ITS1 and ITS4 demonstrated that isolates NC2 and VA17b were *Juglanconis* species. (Accession Number KY427155.1; E-value = 0; 93% identity) [68].

### Developing and identifying polymorphic microsatellite loci

The final genomic assembly spanned 49 Mb with scaffold N50 of 126.6 kb (111 scaffolds) and contig N50 of 33,311 bp (429 contigs). A total of 23,334 sequences produced 2,560 perfect microsatellite loci (933 di-, 1462, tri-, and 165 tetra- nucleotide repeat motif). Forty-seven (18 di- and 29 tri- nucleotide repeat motif) out of 50 primer pairs were optimized to successfully amplify DNA and consistently produce a single product per locus (Table 2). Three primers that failed to provide reliable amplification of a single band were excluded from further analyses. Binning the length of amplicons produced by the primers yielded distinct allelic classes that ranged from one to five alleles per locus. The differences in the size range of alleles in most loci were generally small, typically less than 10 bp. However, eight loci had ranges equal to or greater than 10 bp (Table 2). Ten microsatellite loci with tri-repeat motifs amplified unique alleles that were found only in the pre and post 1993 west populations (DD01, DD03, DD05, DD10, DD015, DD16, DD17, DD018, and DD23, and DD24).

### Population diversity

Clone-correction removed 13 individuals from the original data set (n = 93): 3 from the pre-1993 North, 2 from the pre-1993 South, one from the post-1993 North, 6 from the post-1993 South, and one from the post-1993 West subpopulation. Clone-corrected data (80 haploid individuals with 69 MLGs) was used in all subsequent analyses with i) 47 SSRs that include both polymorphic (n = 40) and monomorphic (n = 7), and ii) only the 40 polymorphic microsatellite loci. Here, we present data only for 47 microsatellite loci because data for 40 loci produced similar results (data not shown).

*Discula destructiva* isolates included in the study were assigned to one of six subpopulations based on common geographical region (North, South, and West) and year of isolation (pre-1993 and post-1993). The mean number of effective alleles (N_e_) per subpopulation ranged from 1.03 in the post-1993 North subpopulation to 1.53 in the pre-1993 West population. Shannon-Weiner index of multilocus genotype (MLG) diversity (H) ranged from 1.1 in post-1993 North, to 3.47 in the pre-1993 South. Genotypic diversity corrected for sample size (H_exp_) across all subpopulations was 0.18, ranging from 0.03 in the post-1993 North to 0.31 in the pre-1993 West (Table 3). Twelve private alleles (Pa) were discovered in five of the six designated subpopulations (Table 3); no private alleles were detected in the post-1993 North subpopulation. The standardized index of association (${\bar{r}}_{d}$ an unbiased measure of linkage disequilibrium) differed significantly from zero, supporting the hypothesis that *D*. *destructiva* reproduces asexually via conidia (Table 3). Moreover, ${\bar{r}}_{d}$ was significantly different from zero across all subpopulations of *D*. *destructiva* except post-1993 North and West, which implies that this fungus reproduces exclusively in an asexual or clonal fashion (S1 Fig). Negative values of ${\bar{r}}_{d}$ were observed in post-1993 North subpopulations, however index of association can be affected by limited number of individuals, which was relatively small for both subpopulations (Table 3).

**Table 3:** Summary information on *Discula destructiva* isolates from six sub-populations (three geographic regions (north, south, and west) and two time periods (pre-1993 and post-1993)) using 47 microsatellite loci.

Population Name	N	N_CC_/MLG	N_e_	H	Pa	H_exp_	${\bar{r}}_{d}$	*P*-value (${\bar{r}}_{d}$)
Pre-1993 North	21	18	1.43	2.89	2	0.27	0.11	<0.01
Pre-1993 South	34	32	1.45	3.47	5	0.27	0.14	<0.01
Pre-1993 West	10	10	1.53	2.3	2	0.31	0.17	<0.01
Post-1993 North	4	3	1.03*	1.1	0	0.03	-0.50*	NS
Post-1993 South	19	13	1.22	2.56	1	0.14	0.25	<0.01
Post-1993 West	5	4	1.09*	1.39	2	0.07	0.17*	0.42
**Total**	**93**	**80/69**						

N—total number of samples for the entire data set; N_CC_ /MLG number of haploid individuals after clone-correction/MLG—number of multi locus genotypes observed after clone correction; N_e_—number of effective alleles; H—Shannon-Wiener index of MLG diversity; Pa—number of private alleles in each population; H_exp_—Nei's genotypic diversity corrected for sample size; ${\bar{r}}_{d}$—the standardized index of association.

*Insufficient sample size to permit accurate interpretation.

### Population structure and genetic differentiation

**Fig 1:**
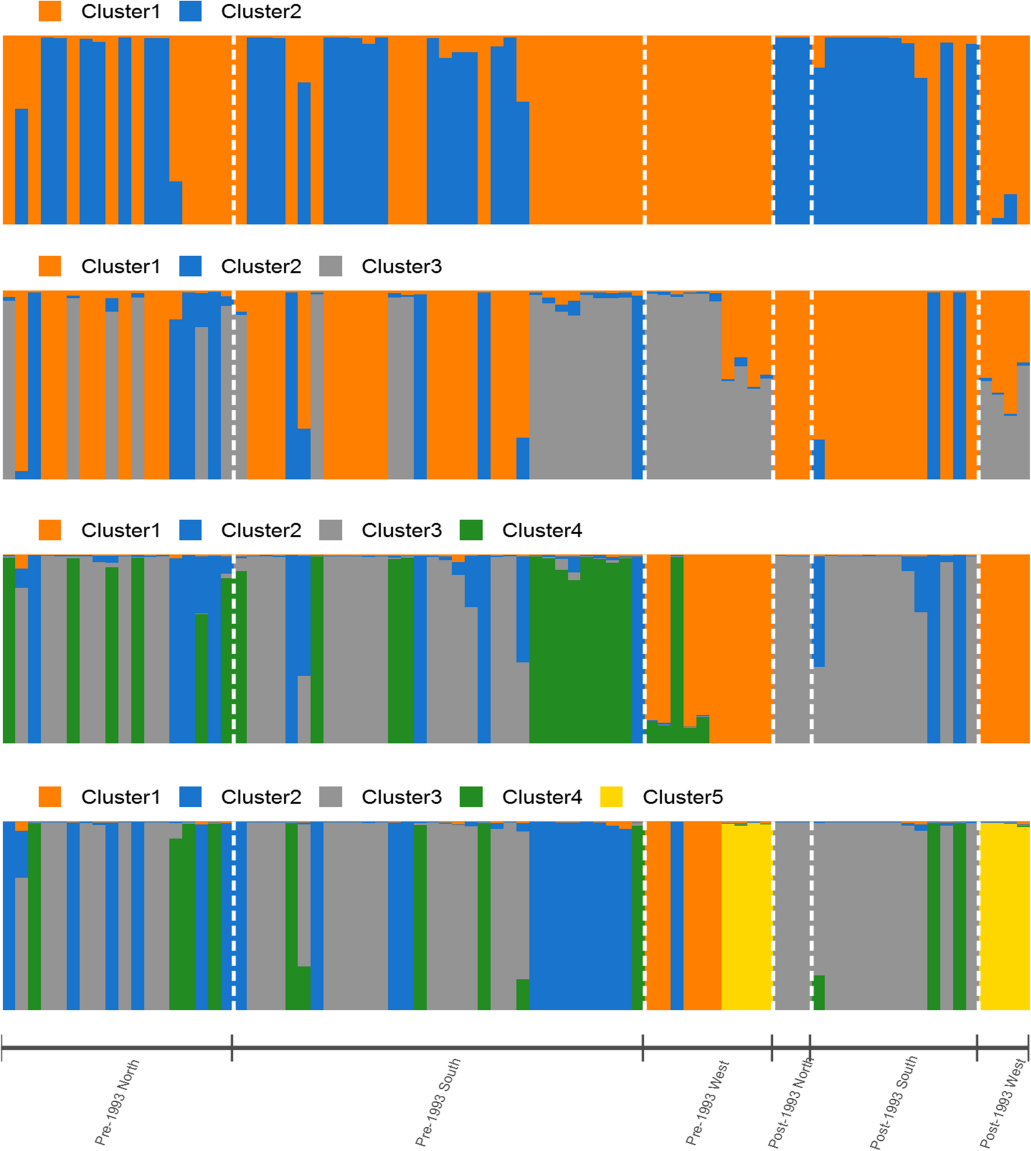
Structure bar graph representing genetic clusters (K = 2–5) of *Discula destructiva* isolates from three geographic regions (north, south, and west) and two time periods (pre-1993 and post-1993) using 47 microsatellite loci. Each bar represents an individual isolate and colors code the proportion of membership of each isolate belonging to one of the designated clusters. Evanno's method indicated that four genetic clusters (K = 4) is the best fitting model.

The presence of four genetic clusters (ΔK = 4) best explains the population structure of *D*. *destructiva* subpopulations across different time periods and geographical regions of collection (Fig 1, S2 Fig). The isolates from the pre-1993 North and South subpopulations had a higher level of admixture when compared to post-1993 and North and South subpopulations (Fig 1). The post-1993 West subpopulation formed a homogenous cluster, and the pre-1993 West subpopulation exhibited comparatively lower levels of admixture (Fig 1). Only one individual assigned to the pre-1993 West cluster had majority assignment probability in the pre-1993 North and South subpopulations (Fig 1). When ΔK = 5 was considered as a possible clustering option for these subpopulations, the pre-1993 West subpopulation was less admixed with pre-1993 North and pre-1993 South subpopulations when compared to the results obtained using four clusters (Fig 1). Under scenario of five clusters, the pre-1993 West subpopulation grouped in part with the post-1993 West cluster.

The analysis of molecular variance (AMOVA) was performed by grouping *D*. *destructiva* subpopulations into the following: i) one hierarchical group, ii) two time periods (pre-1993 and post-1993), iii) two geographic regions (east and west), iv) three groups based on different *D*. *destructiva* hosts–*C*. *florida*, *C*. *nuttallii*, and *C*. *kousa*, and v) four clusters identified by program STRUCTURE. When data were analyzed as one hierarchical group, the majority of variation was within six *D*. *destructiva* subpopulations (70.90%) rather than among sampling locations (29.10%, *P*<0.01) (Table 4). When partitioned based on different time periods (pre- 1993 and post-1993), 10.96% of variation was attributed to variations among the groups from two time periods (*P*<0.01). However, the majority of variation was found within *Discula* groups from these two time periods (89.04%, *F*st = 0.11, *P*<0.01) (Table 4). Furthermore, when data were partitioned based on geographical origin of *D*. *destructiva* subpopulations (eastern and western North America), the majority of the variation was found among individuals within six *Discula* subpopulations (52.75%, *F*st = 0.47, *P*<0.01), whereas 5.40% of variation was explained by differences among *Discula* subpopulations within eastern and western groups (*F*sc = 0.09, *P*<0.01). Although more than 42% of variation was identified between these two geographical regions, the differences were not significant (41.85%, *F*ct = 0.41, *P* = 0.06). When partitioned by the host, 39.21% and 60.79% of the variation was attributed among three host groups and within these groups, respectively (Table 4). However, *C*. *florida* individuals were overrepresented in this dataset and the results pertaining to host information should be interpreted with caution. Lastly, when AMOVA analyses was performed based on four clusters identified by STRUCTURE, majority of variation was attributed to differences among clusters rather than individually based (66.13% and 33.87%, *P*<0.01) (Table 4).

**Table 4:** Analysis of molecular variance (AMOVA) for *Discula destructiva* isolates across 47 microsatellite loci and six subpopulations. Five analyses were conducted—the first included all subpopulations as one hierarchical group (i), the second was based on two time periods—before and after 1993 [ii], the third one was partitioned based on two geographic regions—eastern and western United States (iii), the fourth one was based on three hosts of *D*. *destructiva*, *C*. *florida*, *C*. *nuttallii*, *and C*. *kousa* (iv), and the last one was based on 4 clusters identified by program STRUCTURE (v).

Variance Partition	df	Sum of Squares	Variance Component	% of Variation	*P* value
**(i)**					
Among *Discula* sampling locations	5	150.04	2.09	29.10	*P*<0.01
Within 6 *Discula* subpopulations	74	377.51	5.10	70.90	
Total	79	527.55	7.19		
*F*st = 0.29					
**[ii]**					
Among *Discula* groups from two time periods	1	29.93	0.79	10.96	*P*<0.01
Within *Discula* groups from two time periods	78	497.62	6.38	89.04	
Total	79	527.55	7.15		
*F*st = 0.11					
**[iii]**					
Among eastern and western *Discula* groups	1	104.28	4.04	41.85	*P* = 0.06
Among subpopulations within eastern and western *Discula* groups	4	45.77	0.52	5.40	*P*<0.01
Within 6 *Discula* subpopulations	74	377351	5.1	52.75	*P*<0.01
Total	79	527.55	9.67		
*F*ct = 0.41, *F*sc = 0.09, *F*st = 0.47					
**(iv)**					
Among three host groups	2	108.53	3.51	39.21	*P*<0.01
Within three host groups	77	419.02	5.44	60.79	
Total	79	527.55	8.95		
*F*st = 0.39					
**(v)**					
Among 4 clusters	3	310.34	5.58	66.13	*P*<0.01
Within each of 6 subpopulations among 4 clusters	76	217.21	2.85	33.87	
Total	79	527.55	8.44		
*F*st = 0.66					

*F*ct—the variance among groups relative to the total variance; *F*sc the variance among subpopulations within groups; *F*st—the variance among subpopulations relative to the total variance.

**Fig 2:**
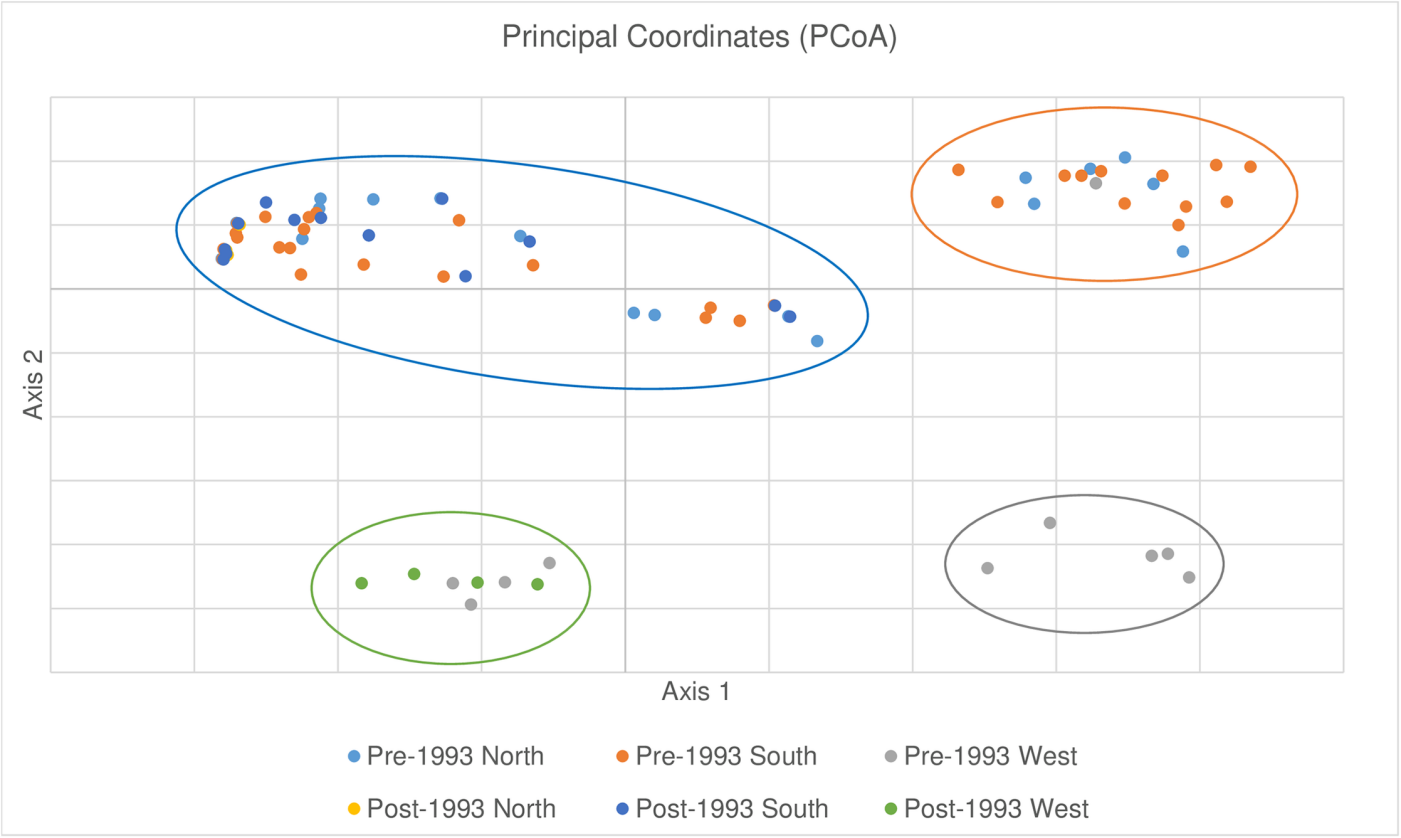
Principle coordinates analysis (PCoA) observed among *Discula destructiva* isolates from three geographic regions and two time periods using 47 microsatellite loci. The first two axes explain 83.51% of the observed variation.

**Fig 3:**
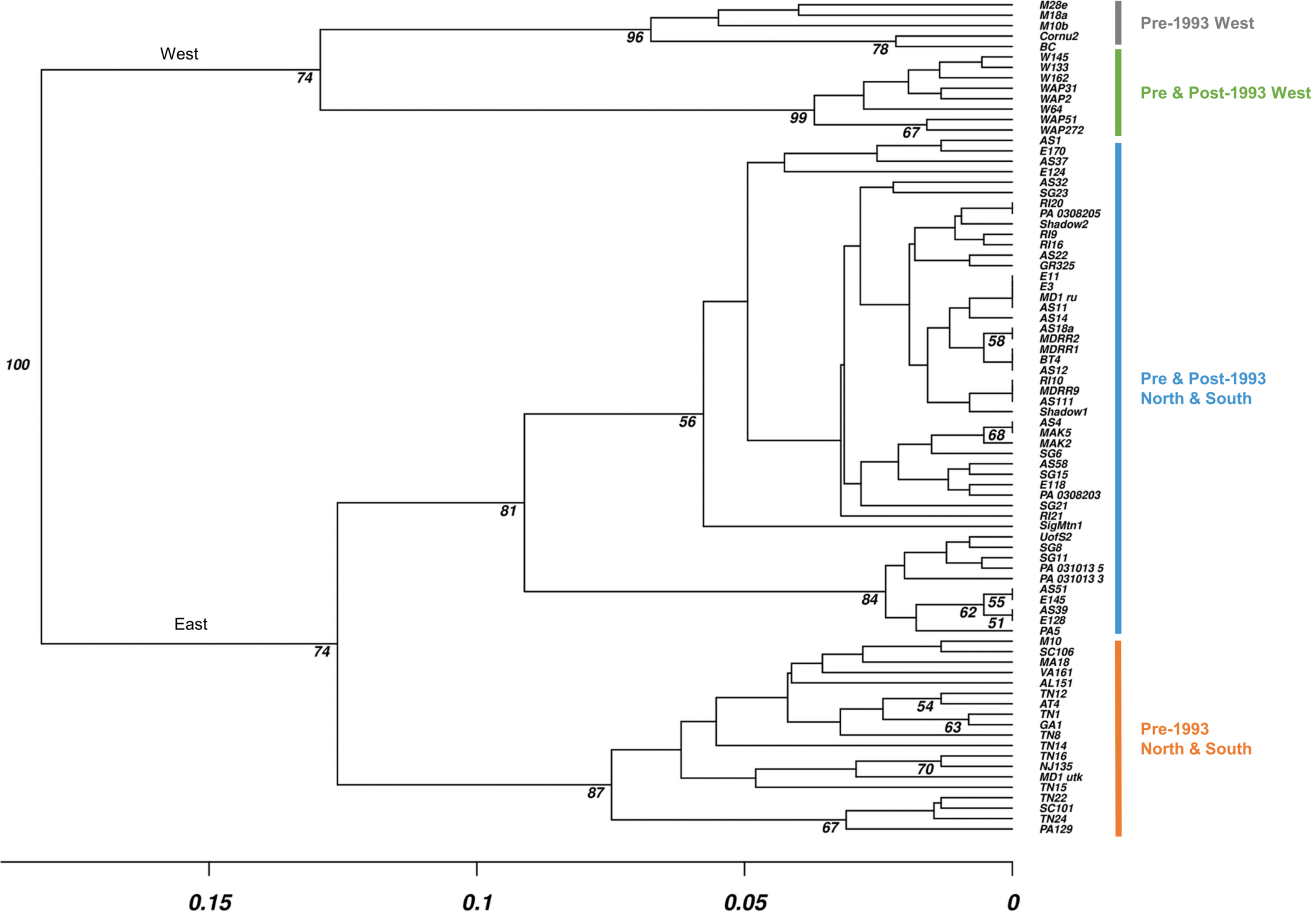
UPGMA dendrogram of *Discula destructiva* isolates collected from three geographic regions and two time periods. Bruvo’s distance was utilized to construct and visualize UPGMA algorithms using 1,000 bootstraps with support values greater than 70%.

Pairwise population differentiation (ϕ_PT_) indicated significant (*P*< 0.05) subpopulation differentiation except between the North and South subpopulations, and ranged from 0.09–0.88 (S1 Table). The majority of differentiation was observed among eastern and western regions. The highest gene flow was detected between pre and post-1993 North and South subpopulations in the eastern U.S, whereas low levels were observed among eastern and western groups (S2 Table). Principle coordinates analysis (PCoA) supported STRUCTURE results by indicating four distinct clusters in which the first two axes explained 83.51% of the observed variation (Fig 2). The pre-1993 West subpopulation clustered separately (grey cluster), whereas individuals from pre and post-1993 West grouped together (green cluster) (Fig 2). Pre-1993 North and South subpopulations grouped together (orange cluster), although one isolate from the pre-1993 western population clustered with the pre-1993 North and South group. The final cluster consisted of individuals from pre and post-1993 North and South (blue cluster). The UPGMA dendrogram revealed the same pattern as shown by the PCoA (S3 Fig). The pre-1993 West isolates formed distinct clusters and pre- and post-1993 West isolates grouped together. The pre- and post-1993 North and South isolates formed a single cluster and the pre-1993 North and South isolates formed a cluster (Fig 3, S3 Fig).

### Historical demography

The program BOTTLENECK was used to determine if a recent population bottleneck or expansion occurred. The Sign tests (Table 5), the Wilcoxon test and the standardized differences tests were applied to all three mutation models, and the results showed significant genetic diversity excess (*P* < 0.01) in all four genetic clusters, which indicated that a recent population bottleneck has occurred. The mode-shift indicator showed allele frequency distortion for all genetic clusters (Table 5).

**Table 5:** Bottleneck determination by sign tests for *Discula destructiva* isolates using 47 microsatellite loci and grouped by the four genetic clusters identified by STRUCTURE.

	Mutation model (excess/deficit)^a^				
Genetic Cluster	I.A.M	T.P.M.	S.M.M	Mode-shift^b^	*P*-value
Cluster 1*	46/0	46/0	46/0	Shifted	*P*<0.01
Cluster 2*	46/0	46/0	46/0	Shifted	*P*<0.01
Cluster 3	47/0	47/0	46/1	Shifted	*P*<0.01
Cluster 4	47/0	47/0	46/1	Shifted	*P*<0.01

I.A.M. = infinite allele model; T.P.M. = two-phase mutation model; S.M.M. = stepwise mutation model.

^a^ Excess/deficit indicates the number of loci showing excess/deficit of gene diversity under mutation-drift equilibrium

^b^ A shift in the distribution of allelic frequency classes is expected in populations that experienced a recent bottleneck. All calculations were based on 10,000 replications.

*One monomorphic locus.

### Cross-amplification of *D*. *destructiva* microsatellite loci across *Discula s*pecies

Seventeen of forty-seven primer pairs were demonstrated to successfully cross amplified DNA from iother *Discula* and *Juglanconis* species (Table 6). An average of 4.6 loci were amplified from *D*. *umbrinella* isolates collected from *Quercus robur* (pedunculate oak) and *Fagus sylvatica* (beech), whereas only 4 loci amplified from *D*. *umbrinella* isolates obtained from *Castanea* (*Ct*.) *sativa* (chestnut). An average of 1.3 loci were amplified from isolates of *D*. *quercina* from *Q*. *garryana* (Oregon white oak) and *C*. *florida*, *D*. *fraxinea* from *Fraxinus americana* (white ash), and *D*. *campestris* from *Acer saccharum* (sugar maple). Four primer pairs amplified loci from isolate NC2, *Juglanconis* sp. (formerly *Melanconis* species) [68], whereas with another *Juglanconis* sp. isolate, VA17b, no loci were amplified. Amplification was observed for an average of 3.8 *Discula* species isolates among the 17 *D*. *destructiva* primer pairs. The greatest number of *Discula* species isolates to which *D*. *destructiva* microsatellites loci were cross-amplified was 12 (DD25), whereas there were several primers (DD15, DD37, and DD42) that only amplified DNA from one other *Discula* species isolate.

**Table 6:** Cross-amplification of seventeen microsatellite loci from *Discula destructiva* across *Discula* spp. and *Juglanconis* species.

	DD02	DD04	DD06	DD08	DD13	DD15	DD22	DD25	DD26	DD30	DD32	DD36	DD37	DD42	DD46	DD47	DD49
Species/ Isolate No.	Amplicon Size																
*Discula umbrinella*																	
67	-^a^	-	-	-	185	-	-	162	169	-	382	-	163	-	-	-	-
115	-	149	-	-	-	-	-	-	-	205	-	-	-	-	188	-	-
116	-	-	204	136	-	-	-	-	-	205	-	-	-	-	188	-	-
221	-	-	-	136	-	-	-	162	169	295	382	146	-	-			*
319	-	-	-	139	-	-	-	162	169	-	382	-	-	-	-	-	-
324	-	-	-	-	-	-	-	162	169	-	382	-	-	-	-	-	*
416	-	-	-	136	-	-	-	-	-	-	-	-	-	-	-	-	*
427	-	-	-	-	-	-	-	-	-	205	-	-	-	-	-	-	-
510	-	-	-	-	-	-	-	162	169	-	382	144	-	-	-		197
518	-	-	-	-	-	*	-	162	169	-	382	144	-	-	-	172	221
611	-	-	-	-	-	173	-	162	169	-	382	144	-	-	-	-	*
DU617	-	-	-	-	-	-	-	162	169	-	382	144	-	-	-	-	-
DUP4	-	-	-	-	-	-	-	162	169	-	382	144	-	-	-	-	-
LT135	-	-	-	-	-	-	166	162	169	-	382	-	-	-	-	-	164
LT068	-	-	-	-	-	-	-	162	180	-	382	-	-	-	-	-	-
*D*. *quercina*	-	-	-	-	-	-	-	-	-	-	-	-	-	-	-	-	-
DQB	-	150	-	-	-	-	-	-	-	-	-	-	-	-	-	-	-
LoMTA	-	-	-	139	-	-	-	-	-	-	-	-	-	-	-	-	-
*D*. *campestris*	-	-	-	-	-	-	-	-	-	-	-	-	-	-	-	-	-
*D*. *fraxinea*	-	-	-	136	-	-	-	-	-	-	-	199	-	-	-	-	-
*Juglanconis* sp.	-	-	-	-	-	-	-	-	-	-	-	-	-	-	-	-	-
VA17b	-	-	-	-	-	-	-	-	-	-	-	-	-	-	-	-	-
NC2	161	-	-	-	-	-	-	181	-	-	-	121		143	-	-	-
D. destructiva^b^	162	150	197	136–139	178–186	129–153	192–197	179–185	179–189	199–203	157–160	195–200	160–163	146–152	188–193	167–172	154–160

a no amplification product

* indicates multiple amplicons present

b range of amplicon base pairs from Table 2

## Discussion

The results of our study indicated significant population structure, low genetic diversity with limited gene flow, and high genetic differentiation among *D*. *destructiva* subpopulations from different geographical regions and two time periods. We also detected signatures of a recent population bottleneck and evidence of asexual reproduction among most *D*. *destructiva* subpopulations. The low genetic diversity among *D*. *destructiva* isolates found here supports previous studies that utilized DAF [35], ASAP [36], and AFLPs [40]. The current assessment of diversity among isolates lends support to the earlier hypothesis that *D*. *destructiva* is an exotic species, which was recently introduced into east and west coasts of North America [14, 16, 25, 35, 40]. In our study, pre-1993 subpopulations had higher diversity when compared to post-1993 subpopulations. However, the steepest decline was observed among pre- and post-1993 North subpopulations, although our limited sample size can produce biased results.

Using Bayesian approaches, *D*. *destructiva* exhibited significant population structure represented by four genetic clusters that correspond to historical and geographical distribution of the pathogen and times of collection. Genetic clusters defined by time of collection (pre- and post-1993) can be explained by multiple introductions of the pathogen occurring on each coast of North America. Distinct genetic clusters and population differentiation separated eastern and western isolates, which corroborates the findings of previous *D*. *destructiva* studies [36, 38–40], and supports the scenario of separate introductions on the east and west coasts.

Our analysis indicated that 29% of genetic differentiation was attributed to variance among *D*. *destructiva* sampling localities; the majority of variation is individually based (71%) (*P*<0.01). Approximately 89% and 53% of variance was identified within subpopulations between two time periods (*P*<0.01) and different geographical origin (*P*<0.01), providing evidence that the majority of differentiation was individually based, rather than among these groups. Although our data indicated that 42% of variation could be explained by eastern and western groups, the results were not significant and could be explained by unequal sample size in our dataset. When grouped by host species, 39% of variation was attributed to the differences among three host groups (*P*<0.01). However, these results should be interpreted with caution because the majority of individuals used in our study were collected from *C*. *florida*, possibly resulting in biased estimations. When *D*. *destructiva* subpopulations were grouped based on STRUCUTRE findings, the majority of genetic variation was found among the clusters (66%, *P*<0.01). Only 34% of variation could be explained by variation within each of the six subpopulations among the four identified clusters.

According to the Millennium Ecosystem Assessment [69], one of the five major drivers of the loss of biodiversity and changes in ecosystem services is due to invasive exotic species. As international trade increases, so does the introduction of new pathogens and pests into new areas and onto new hosts [70]. Introduced forest pathogens account for the loss of $2.1 billion in forest products each year in the U.S.A., although the true environmental costs cannot be measured [71, 72]. Human activity, especially globalized trade, provides a pathway for the introduction of potential pathogens and pests that can have devastating ecological, social, and financial impacts [73–75]. Native trees are threatened mostly by introduced organisms, fungi in particular [70, 73]. Plant diseases can be so devastating that the impacts not only influence biodiversity and ecosystems, but human history as well: for example, the Irish potato famine caused by *Phytophthora infestans* that resulted in millions of people either being forced to migrate or die from starvation [75, 76]. Understanding the pathways by which foreign pathogens, like *D*. *destructiva*, are introduced is vital to the prevention of future invasions and potentially devastating consequences [74].

Low genetic diversity is commonly found in introduced species because of founder effects, in which a small number of representatives, or even a single genotype of the organism, becomes the founder(s) of a population in the new area. Higher genetic diversity would be expected if this species were native to North America [77, 78]. In contrast, *D*. *umbrinella* that is indigenous to Europe had high genetic variability among isolates as measured with RAPDs [79]. Low levels of diversity found among *D*. *destructiva* isolates in previous studies [34–36] suggested that a population bottleneck, or a significant reduction in population size, may have occurred, which was confirmed in our study. The distortion uncovered by the mode-shift indicator occurs when fewer alleles are present at low frequency and shifted into intermediate frequency allele classes, which is a characteristic of recent population bottlenecks [80]. These results support the hypothesis of a founder effect, in which limited genetic diversity in a population is due to a small number of representatives from a more diverse native population being introduced into a new geographical area [77, 78].

Geographical barriers, climatic factors, and the great distances that separate these genetic clusters can be a plausible explanation for limited gene flow and divergence among *D*. *destructiva s*ubpopulations. The lack of gene flow between east and west coast isolates indicated that *D*. *destructiva* did not spread from eastern North America to western North America or vice versa. *Discula destructiva* isolates from pre- and post- 1993 in North and -South showed significant gene flow that suggested multiple independent introductions into these areas. However, due to limited and unequal sample size in some subpopulations, we cannot exclude the bias in our migration assumptions. This issue can be mitigated with a larger collection of data that includes the source population, which is believed to be predominantly in Japan and to some extent in China [41]. In organisms with limited gene flow, newly established founder populations are likely to have reduced genetic diversity [81], which was observed across all *D*. *destructiva* subpopulations. Nevertheless, a single pre-1993 West isolate (M10 from UTK), clustered with the pre-1993 eastern group (both North and South), which suggests the possibility of movement between coasts, probably due to the importation of infected trees from the western to the eastern U.S.A. The introduction of dogwood anthracnose into Missouri and Kansas was reported to have occurred due to the movement of infected nursery stock [23], demonstrating that inter-state trade has resulted in transporting dogwood anthracnose within North America.

An exotic fungus may not exhibit a high degree of virulence on its native host, but when it comes in contact with a new host, the fungus can become extremely virulent because of the lack of host resistance that develops over time through coevolution [73]. This could be the case with *D*. *destructiva* and the native dogwood species of North America [41]. Understanding how a new disease spreads is important when implementing preventive measures and control strategies [73]. Dogwood anthracnose is an example of how an introduced fungal pathogen can spread quickly over the geographic range of the new host and reach epidemic proportions. Analyzing the population structure and genetic diversity of a pathogen helps to uncover the history of the pathogen, allowing insight into its origin, how it entered the new area, and potential consequences [82].

The standardized index of association (${\bar{r}}_{d}$) was used to determine if populations reproduce asexually or sexually by examining multilocus linkage disequilibrium. Significant linkage disequilibrium (*P* < 0.05) was present among *D*. *destructiva* isolates due to linkage among loci indicating asexual or clonal reproduction [51]. The low genetic diversity and asexual reproduction of *D*. *destructiva* are indicative of an introduced pathogen [83]. Although a negative ${\bar{r}}_{d}$ value was observed, the value might have been affected by the limited sample size, which was reduced to three individuals after clone-correction of that data. Other subpopulations provided significant departure from zero, indicating presence of clonal reproduction among *D*. *destructiva*. Our findings may suggest that sexual reproduction of *D*. *destructiva* may have occurred infrequently in the past, although it has never been observed in a laboratory or natural environment in North America. In fact, the complete lack of sexual reproduction has led investigators to propose conservation of the imperfect or asexual stage (*D*. *destructiva*) as the permanent scientific name [33].

Redlin [25] provided the scientific description of *D*. *destructiva*, which was previously known as *Discula* Type I. He noted that some fungi isolated from diseased tissues resembled *D*. *destructiva*, but had sufficiently different colony and conidiophore morphological that they could not be assigned to this newly described species. As a result, they were referred to as *Discula* Type II [14, 30]. Additionally, all *D*. *destructiva* isolates produced polyphenol oxidase, whereas the morphological variants did not [30]. Sequencing of the ITS region with primers 1 and 4 or 1 and 2 [45] confirmed that the Type I isolates were *D*. *destructiva*. Furthermore, the two Type II isolates, NC-2 and VA17b included in this study and another isolate (NY326) described in Trigiano et al. (1995) were identified as *Juglanconis* species (formerly *Melanconis* species) [68], which are pathogens of birch and closely related to some other *Discula* species [31, 84]. Both genera occupy the same clade in the Diaporthales, but some *Discula* species, including *D*. *destructiva*, are classified in the Gnomoniaceae, whereas, *Juglanconis* species are placed in Juglanconidaceae [68]. A BLAST search using the ITS sequences from other *Discula* species included in this study confirmed their identity as *Discula*.

The test for cross-amplification of the microsatellite primer pairs revealed shared loci between *D*. *destructiva* and other *Discula* species, indicating they were related, and thus, allowed their relationships to be analyzed [85, 86]. Based solely on the number of shared primer pairs that cross-amplified, *D*. *destructiva* appears to be most closely related to *D*. *umbrinella* isolates from oak and beech, followed by *D*. *umbrinella* from chestnut. *Discula quercina*, *D*. *fraxinea*, and *D*. *campestris* are less closely related to *D*. *destructiva*, with the *Juglanconis* species being more distantly related. This relationship is similar to that described in a previous study [84], but *D*. *umbrinella* was not included in their analyses. The UPGMA strongly supported the divergence of *D*. *destructiva* from other *Discula* species and found similar genetic relationships to those revealed by previous studies in which eastern and western *D*. *destructiva* isolates were separated from each other [36, 40] and from other *Discula* species [34]. Moreover, *D*. *destructiva* loci (DD25, DD26, and DD32) amplified DNA from most *D*. *umbrinella* isolates and are probably suitable for studying genetic diversity and population genetics of this related species. However, because of an insufficient sample size of other *Discula* and *Juglanconis* species isolates and the apparent lack of the same microsatellite loci, we cannot study the genetic diversity and population genetics of these other species.

In conclusion, the 47 microsatellite primer pairs used in this study successfully amplified loci in *D*. *destructiva* and other *Discula* species. Sufficient allelic data were produced by these primers to analyze genetic diversity, population structure, genetic differentiation and linkage disequilibrium of *D*. *destructiva* isolates. *Discula destructiva* has been considered an introduced pathogen because of the sudden appearance of dogwood anthracnose near ports of entry on both the east and west coasts of North America, which rapidly spread across the distribution of native dogwoods and caused significant losses of trees. The results of our study support this scenario and provide a new evolutionary perspective that may be valuable in future analyses and models. Future population studies involving Asian specimens could elucidate the origin of *D*. *destructiva* and provide novel insight into the spread and distribution of this invasive pathogen.

## Supporting information

S1 Table Pairwise population differentiation for *Discula destructiva* isolates from three geographic regions and two time periods using 47 microsatellite loci. (DOCX)

S2 Table Pairwise gene flow values for *Discula destructiva* isolates from three geographic regions and two time periods using 47 microsatellite loci. (DOCX)

S1 Fig Visualization of linkage disequilibrium tests of 80 *Discula destructiva* isolates using 47 microsatellite loci. Data was obtained after 10,000 permutations.
(TIF)

S2 Fig Maximum DK at K = 4 for *Discula destructiva* isolates using 47 microsatellite loci. (TIF)

S3 Fig Dendrogram of *Discula destructiva* isolates collected from three geographic regions and two time periods. Nei's genetic distance was utilized to construct and visualize phylogenetic tree using 1,000 bootstraps with support values greater than 50%.
(TIF)
