# Supplementary material for: Confirmation of independent introductions of an exotic plant pathogen of Cornus species, Discula destructiva, on the east and west coasts of North America
Source: PLoS One. 2017 Jul 26;12(7):e0180345. doi: 10.1371/journal.pone.0180345 (PMC5528261; doi:10.1371/journal.pone.0180345)
Supplement: S1 Table — (DOCX) [file pone.0180345.s001.docx]

| S1 Table. Pairwise population differentiation for *Discula destructiva* isolates from three geographic regions and two time periods using 47 microsatellite loci. | | | | | | |
| --- | --- | --- | --- | --- | --- | --- |
|  |  |  |  |  |  |  |
|  | | | | | | |
|  | **Pre-1993 North** | **Pre-1993 South** | **Pre-1993 West** | **Post-1993 North** | **Post-1993 South** | **Post-1993 West** |
| **Pre-1993 North** | 0 |  |  |  |  |  |
| **Pre-1993 South** | 0* | 0 |  |  |  |  |
| **Pre-1993 West** | 0.39 | 0.40 | 0 |  |  |  |
| **Post-1993 North** | 0.19 | 0.17 | 0.51 | 0 |  |  |
| **Post-1993 South** | 0.09 | 0.09 | 0.54 | 0.07* | 0 |  |
| **Post-1993 West** | 0.54 | 0.53 | 0.28 | 0.88 | 0.69 | 0 |
|  | | | | | | |
| *F*st values are based on 10,000 permutations; significant values *P*<0.05, *ns=not significant. | | | | | | |
