# Supplementary material for: Confirmation of independent introductions of an exotic plant pathogen of Cornus species, Discula destructiva, on the east and west coasts of North America
Source: PLoS One. 2017 Jul 26;12(7):e0180345. doi: 10.1371/journal.pone.0180345 (PMC5528261; doi:10.1371/journal.pone.0180345)
Supplement: S2 Table — (DOCX) [file pone.0180345.s002.docx]

| S2 Table. Pairwise gene flow values for *Discula destructiva* isolates from three geographic regions and two time periods using 47 microsatellite loci. | | | | | | |
| --- | --- | --- | --- | --- | --- | --- |
|  | | | | | | |
|  | **Pre-1993 North** | **Pre-1993 South** | **Pre-1993 West** | **Post-1993 North** | **Post-1993 South** | **Post-1993 West** |
| **Pre-1993 North** | 0 |  |  |  |  |  |
| **Pre-1993 South** | 0.00 | 0 |  |  |  |  |
| **Pre-1993 West** | 0.76 | 0.74 | 0 |  |  |  |
| **Post-1993 North** | 2.03 | 2.42 | 0.47 | 0 |  |  |
| **Post-1993 South** | 4.80 | 4.72 | 0.04 | 6.91 | 0 |  |
| **Post-1993 West** | 0.41 | 0.45 | 1.27 | 0.06 | 0.22 | 0 |
