# Supplementary figures and images for: Confirmation of independent introductions of an exotic plant pathogen of Cornus species, Discula destructiva, on the east and west coasts of North America
Source: PLoS One. 2017 Jul 26;12(7):e0180345. doi: 10.1371/journal.pone.0180345 (PMC5528261; doi:10.1371/journal.pone.0180345)

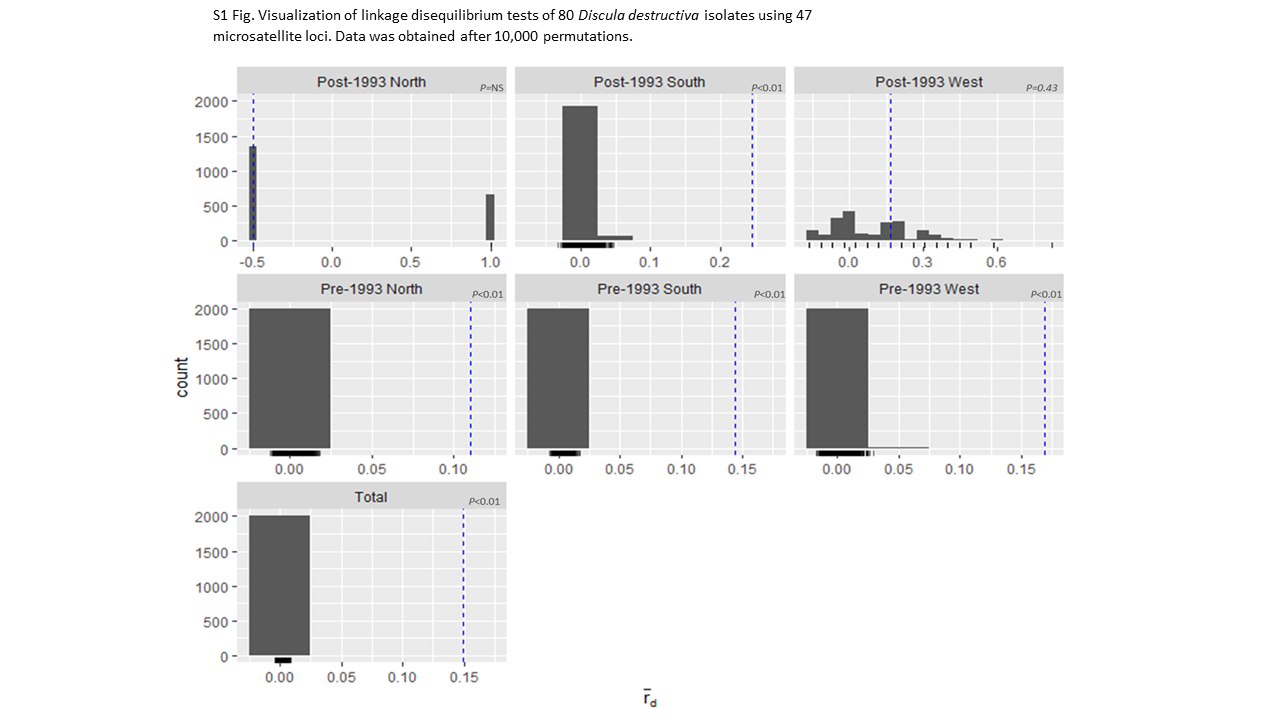

Supplement: S1 Fig — Data was obtained after 10,000 permutations. (TIF) [file pone.0180345.s003.tif]

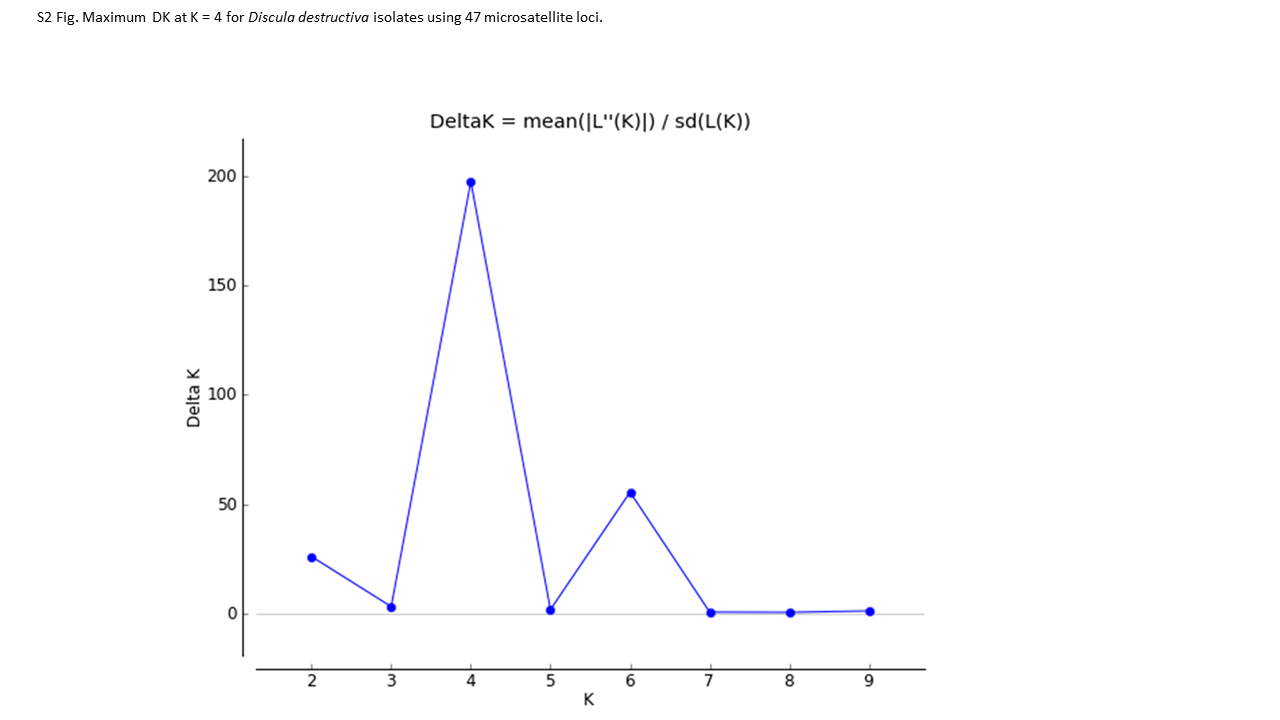

Supplement: S2 Fig — (TIF) [file pone.0180345.s004.tif]

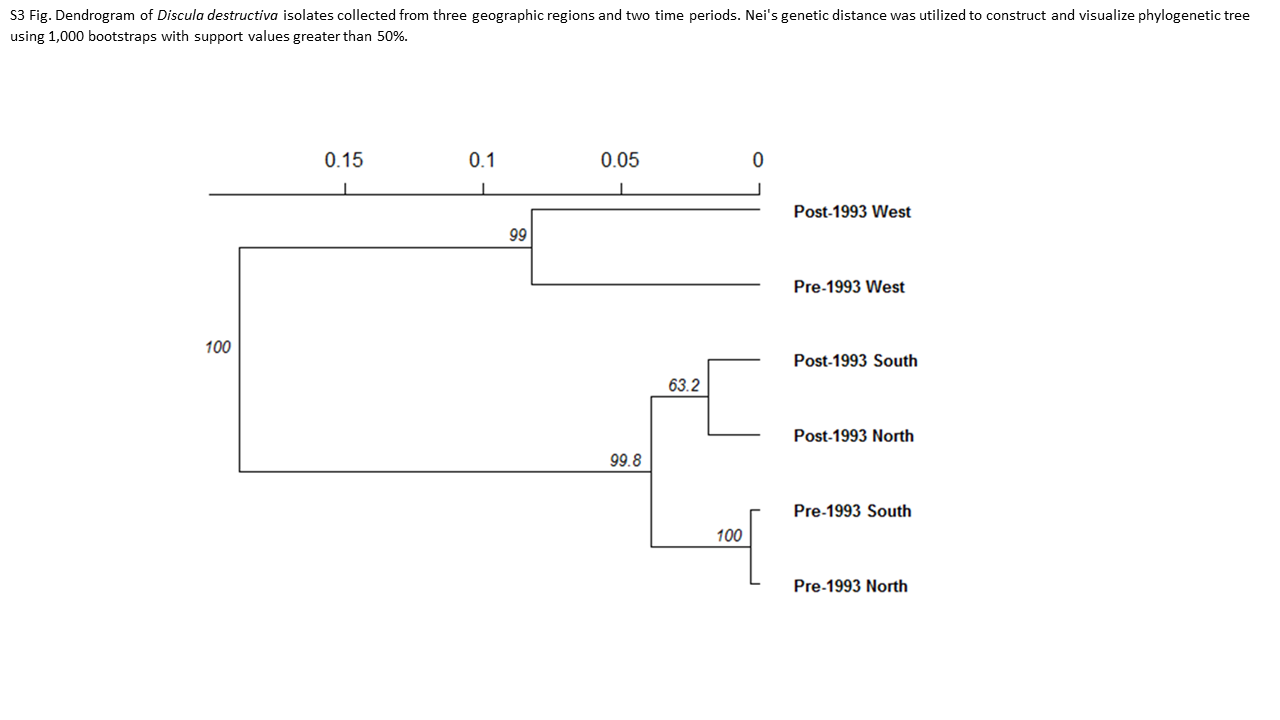

Supplement: S3 Fig — Nei's genetic distance was utilized to construct and visualize phylogenetic tree using 1,000 bootstraps with support values greater than 50%. (TIF) [file pone.0180345.s005.tif]
